# AVP-Induced Increase in AQP2 and p-AQP2 Is Blunted in Heart Failure during Cardiac Remodeling and Is Associated with Decreased AT1R Abundance in Rat Kidney

**DOI:** 10.1371/journal.pone.0116501

**Published:** 2015-02-06

**Authors:** Sophie Constantin Lütken, Jørgen Frøkiær, Søren Nielsen

**Affiliations:** 1 Department of Biomedicine -Anatomy, University of Aarhus, DK-8000 Aarhus C, Denmark; 2 Institute of Clinical Medicine, Aarhus University Hospital, DK-8200 Aarhus N, Denmark; 3 Department of Health Science and Technology, Aalborg University, DK-9220 Aalborg Ø, Denmark; Emory University, UNITED STATES

## Abstract

**Aim:**

The objective was to examine the renal effects of long-term increased angiotensin II and vasopressin plasma levels in early-stage heart failure (HF). We investigated the regulations of the V2 vasopressin receptor, the type 1A angiotensin II receptor, the (pro)renin receptor, and the water channels AQP2, AQP1, AQP3, and AQP4 in the inner medulla of rat kidney.

**Methods:**

HF was induced by coronary artery ligation. Sixty-eight rats were allocated to six groups: Sham (N = 11), HF (N = 11), sodium restricted sham (N = 11), sodium restricted HF (N = 11), sodium restricted sham + DDAVP (N = 12), and sodium restricted HF + DDAVP (N = 12). 1-desamino-8-D-arginine vasopressin (0.5 ng h-1 for 7 days) or vehicle was administered. Pre- and post-treatment echocardiographic evaluation was performed. The rats were sacrificed at day 17 after surgery, before cardiac remodeling in rat is known to be completed.

**Results:**

HF rats on standard sodium diet and sodium restriction displayed biochemical markers of HF. These rats developed hyponatremia, hypo-osmolality, and decreased fractional excretion of sodium. Increase of AQP2 and p(Ser256)-AQP2 abundance in all HF groups was blunted compared with control groups even when infused with DDAVP and despite increased vasopressin V2 receptor and Gsα abundance. This was associated with decreased protein abundance of the AT1A receptor in HF groups vs. controls.

**Conclusion:**

Early-stage HF is associated with blunted increase in AQP2 and p(Ser256)-AQP2 despite of hyponatremia, hypo-osmolality, and increased inner medullary vasopressin V2 receptor expression. Decreased type 1A angiotensin II receptor abundance likely plays a role in the transduction of these effects.

## Introduction

Heart failure (HF) is associated with activation of the renin-angiotensin system (RAS) and sustained increased vasopressin (AVP) release from the pituitary gland [1–5]. RAS and AVP have been shown to play a role in the kidneys by taking part in the development of hyponatremia and water retention. Hyponatremia and water retention in HF is associated with a poor outcome [6,7]. There is increasing evidence of a crosstalk between angiotensin II (ANG II) and AVP with potential enhancing effects on water retention mediated by renal water channels [8–10]. We have previously demonstrated that rats with chronic HF 21 days after myocardial infarction (MI) increased the abundance of the renal water channel aquaporin-2 (AQP2) in the inner medullary collecting ducts (IMCDs) [11]. When treated with the type 1_A_ angiotensin II receptor (AT1R) blocker candesartan, HF rats down regulated IMCD AQP2 expression to sham levels [9]. This supports that a crosstalk between the V2 vasopressin receptor (V2R) and AT1R is possible and potentially important in AQP2 regulation. In contrast, the constitutively expressed AQP1 including the basolateral aquaporins AQP3 and AQP4 remained unchanged both in rats after water loading and in HF rats [11–13].

HF is a progressive condition with short- and long-term adaptations to maintain blood pressure and perfusion to vital organs. Previous HF studies focused on water retention in the stable intermediate stage of HF after 21 days in the rat, when cardiac remodeling has been completed [14–16]. Even though diuretics play a crucial role in standard HF treatment, the subcellular basis for the development of water retention has not previously been investigated in the early stage after MI, a period of clinical interest due to the increased risk of arrhythmias and death [17]. Furthermore, the complexity of HF makes it difficult to distinguish between the various actions of key hormones in HF development. As HF is an evolving condition, one could speculate whether initial and potential beneficial adaptations are abolished in later stages of disease.

Low-sodium diet is a well-known method to increase endogenous ANG II levels. In co-treatment with infusions of the selective V2-receptor (V2R) agonist 1-desamino-8-D-arginine vasopressin (DDAVP), low-sodium diet has been used in rat models to study renal changes in water retention [8,18]. To our knowledge these models have never been directly compared with or applied to a HF model. Thus, the aim of the present study was to 1) investigate whether the inner medullary changes to low-sodium diet and DDAVP infusion in controls are comparable with the changes seen in early-stage HF. 2) Investigate the renal effects of clamped ANG II levels in early-stage HF in combination with DDAVP. 3) to investigate whether IM expressions of AQP2, p-AQP2 and AQP1, AQP3, and AQP4 in early-stage HF are changed compared with healthy animals under basic states and in conditions with enhanced ANG II levels and DDAVP infusion, and 4) to examine whether these changes are associated with changes in cardiac function, V2R, AT1R and the (pro)renin receptor (P)RR.

## Methods

### Experimental animals

Sixty-eight male Munich-Wistar rats obtained from Harlan Laboratories, Denmark with an initial weight of 250 g were initially given free access to tap water and standard rat chow (Altromin 1324, Altromin, Lage, Germany). The rats were housed under controlled temperature (21 ± 2°C) and humidity (55 ± 2%) in a 12:12-h light-dark cycle and acclimatized for 7 days before surgery. All animal protocols were approved by the board at the Institute of Clinical Medicine, University of Aarhus according to the licenses for use of experimental animal issued by the Danish Ministry of Justice.

### Animal preparations

HF was induced by free wall MI following ligation of the left anterior descending artery (LAD) as previously described in detail [9]. Sham operated animals underwent the same procedure, without ligation of the LAD. Buprenorphine (0.12 mg/kg sc; Anorfin, GEA, Frederiksberg, Denmark) was administered twice for 2 days to relieve postoperative pain. The rats were kept in a 100% oxygen environment for 24 h after surgery. With this method mortality rate was 15% in rats that underwent LAD ligation.

### Study design

**Fig 1:**
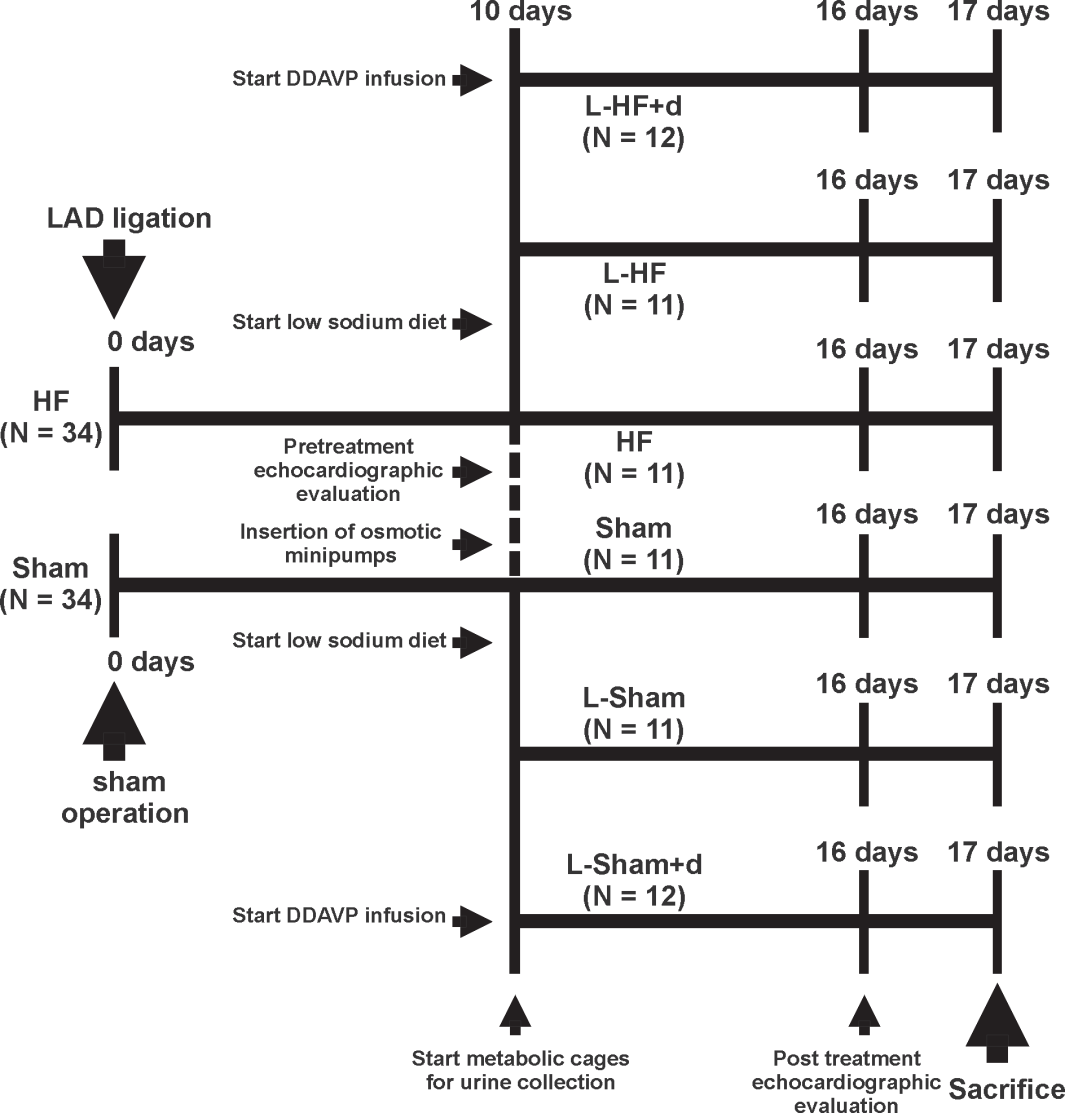
Diagram of study design. Sham operation or ligation of the left anterior descending artery (LAD) was performed day 0. Day 10: The rats underwent echocardiographic evaluation before allocation to standard diet or low sodium diet. Half of the rats on low sodium diet also received DDAVP infusion in osmotic minipumps for 7 days. The rats were maintained in metabolic cages over the last 7 days for clearance studies. At day 16, post-treatment echocardiographic evaluation was performed. Day 17 the rats were sacrificed and the kidneys processed for membrane fractionation and immunoblotting the same day.

As shown in Fig. 1, sham operation or ligation of the LAD was performed day zero (Sham rats, N = 34; HF, N = 34). For increasing survival rate, the animals were allowed to recover the surgical procedure for ten days. At day ten the two groups were subdivided into six groups as described in the following. After echocardiographic evaluation, the rats were allocated into two diet types. Either standard diet (Altromin 1324, Altromin, Lage, Germany) containing 0.2% sodium (Sham rats, N = 11; HF, N = 11), or low sodium diet (Altromin C1036) containing 0.015% sodium (L-Sham, N = 23; L-HF, N = 23). Estimated daily sodium intake in the animals receiving low sodium diet was 0.60 meq Na^+^· (250g BW)^-1^ ·day^-1^. The rats receiving low sodium diet were further subdivided. Thus, half of the rats on low sodium diet received DDAVP infusion (V1005, Sigma, 0.5 ng · h^-1^) dissolved in physiological saline for 7 days administered via osmotic minipumps (Alzet mini-osmotic pumps model 2201, Scanbur, Køge, Denmark), as previously described (L-Sham, N = 11; L-HF, N = 11 and L-Sham+d, N = 12; L-HF+d, N = 12) [8]. All rats not receiving DDAVP were treated with vehicle only. All groups were matched according to ejection fraction (EF) and weight and had free access to tap water during the entire experiment. The animals were placed individually in metabolic cages (Scanbur, Køge, Denmark) for the last seven days of the experiment to allow clearance studies. After seven days of DDAVP infusion and seventeen days after MI, the rats were sacrificed and the kidneys were rapidly removed and processed for membrane fraction and immunoblotting the same day.

### Echocardiographic evaluation

Day ten and sixteen in experiment echocardiographic evaluation was performed. Transthoracic echocardiography was obtained with Vivid7 Ultrasound Scanner (GE Medical Systems) using a 10S transducer (11.5 MHz). The echocardiographic scanner was kindly provided by Professor Erik Sloth, Department of Anaesthesia and Intensive Care, Aarhus University Hospital –Skejby, Denmark. All images and subsequent measurements were performed according to American Society of Echocardiography (ASE) guidelines [19,20]. The rats were lightly anesthetized with 1–2% isoflorane and 100% oxygen, their chest shaved and cleaned with alcohol, and electrode pads with ECG electrodes were gently placed with sleek tape on both forepaws and left hindpaw. Penetration depth was 2 cm, width was as small as possible, and frames per second were set on maximal. The rats were positioned on their left side with the transducer placed on the left hemithorax. Care was taken to avoid pressure on the thorax potentially causing bradycardia and alteration of cardiac filling. Two-dimensional images and M-mode tracings of the parasternal short-axis view at the level of the papillary muscles and apical four-chamber view were obtained. It was ensured that the image was on axis based on roundness of the left ventricular cavity at the level of the papillary muscles. The time of end-diastole was defined as the maximal diameter of the left ventricle (LV). Accordingly, end-systole was defined as the minimal diameter in the same heart cycle. At least three heart cycles were averaged for each measurement. All measurements were performed manually. Data were analyzed using Echopac PC (GE Medical Systems). Left ventricular end-diastolic and end-systolic volumes (LVEDV and LVESV, respectively) were calculated from LV diastolic (LVDA) and LV systolic (LVSA) areas via the bullet equation [21,22]. Ejection fractions (EF) were calculated from diastolic and systolic volumes as [(LVEDV-LVESV)/LVEDV 100]. Heart rate (HR) was calculated from beats per second obtained by pulse wave Doppler mitral inflow measurements from the apical four-chamber view during ten seconds due to the fast heart rate in small animals. Sample volume was set at the highest size available during this particular recording. HR as beats pr. minute (BPM) was calculated as [number of systolic mitral inflows s^-1^ 60]. Inclusion criteria for the rats that underwent LAD-ligation was EF < 50% at day ten in experiment.

### Clearance studies

Before euthanasia, 6–8 ml of blood was collected into a heparinized tube for determination of plasma electrolytes and osmolality. For clearance studies, urine samples were immediately stored at -20°C. Plasma concentrations of sodium and potassium were determined on the last day of experiment (Vitros 950, Johnson & Johnson). The concentrations of urinary sodium and potassium were determined by standard flame photometry (Eppendorf FCM6341). The urine and plasma osmolality was measured with a vapor pressure osmometer (Osmomat 030, Gonotec, Berlin, Germany). From the obtained measurements, electrolyte free water reabsorption (T^e^cH_2_O) was calculated as:

$$T^{e}{\mathrm{cH}}_{2}O=UO\cdot \left(\frac{UNa+UK}{PNa}-1\right)$$

Where T^e^cH_2_O is electrolyte free water reabsorption, UO is daily urinary flow rate, UNa is urine sodium, UK is urine potassium, and PNa is plasma sodium [23–25].

### Immunohistochemistry

After rapid anesthesia with 3% isoflurane, the left kidneys were fixed by retrograde perfusion via the aorta with 3% paraformaldehyde in 0.01 M PBS, pH 7.4. Briefly, the kidney was removed, and the midregion was sectioned into 2- to 3-mm transverse sections and immersion fixed for an additional 1 h, followed by three 10-min washes with 0.01 M PBS buffer, pH 7.4. The tissue was dehydrated in graded ethanol and left overnight in xylene. Paraffin-embedded sections (2 μm thickness) were cut on a rotary microtome (Leica Microsystems, Herlev, Denmark). Immunolabeling with AQP2 and p-AQP2 was performed on sections from the paraffin-embedded preparation using methods described previously in detail [9,26].

### Semiquantitative immunoblotting

The right kidney was quickly removed and inner medulla (IM) dissected from the remaining kidney. IM from each rat was individually homogenized (Ultra-Turrax T8 homogenizer, IKA Labortechnik, Staufen, Germany) in ice-cold isolation solution containing 0.3 M sucrose, 25 mM imidazole, and 1 mM EDTA, pH 7.2, the protease inhibitors 8.5 μM leupeptin (Sigma-Aldrich) and 0.4 mM Pefabloc (Roche) and the phosphatase inhibitors sodium orthovanadate (0.0184 g/100 ml buffer; Sigma-Aldrich), sodium fluoride (0.1052 g/100 ml buffer; Merck, Whitehouse Station, NJ), and okadaic acid (16.4 μl/100 ml buffer; Calbiochem, San Diego, CA). The homogenates were then centrifuged at 1000 g for 15 min at 4°C to remove whole cells, nuclei, and mitochondria, and gel samples were prepared from the supernatant in Laemmli sample buffer containing 2% SDS and dithiothreitol and solubilized at at 65°C, and stored at -20°C. The total protein concentration of the homogenate was measured using a Pierce BCA protein assay kit (Roche, Basel, Switzerland). Samples of membrane fractions were run on polyacrylamide gels (Criterion TGX Long Shelf Life Precast Gels, Any kD, Bio-Rad Laboratories Inc., USA). To ascertain identical loading and to allow for correction, an identical gel was run in parallel and subjected to Coomassie Brilliant Blue staining, as previously described in detail [9,27]. SDS-PAGE was performed on polyacrylamide gels (Criterion TGX Long Shelf Life Precast Gels, Any kD and Criterion TGX Precast Gels 4–15%, Bio-Rad Laboratories Inc., USA). Each lane was loaded with ~12 μg of protein from samples from a different rat.

The proteins from kidney IM were transferred to a polyvinylidene difluoride (PVDF) membrane (Immobilon-P PVDF Transfer Membrane, Millipore, Cat. No. IPVH 00010) using the BioRad Trans-Blot Turbo Transfer System with an ice-cold transfer buffer containing 200 ml 5 x Bio-Rad Trans-Blot Turbo Transfer Buffer mixed with 600 ml nanopure water and 200 ml 92% ethanol. The blots were blocked with 5% nonfat dry milk in PBS-T (80 mM Na_2_HPO_4_, 20 mM NaH_2_PO_4_, 100 mM NaCl, 0.1% Tween 20, adjusted to pH 7.4 with 10 M NaOH) or by using a blocking buffer containing 50% 0.1M PBS and 50% Odyssey Infrared Imaging System Blocking Buffer (LI-COR Biosciences, Cambridge, UK). After washing with PBS-T, the blots were incubated with primary antibodies overnight at 4°C on a tilting table. The antigen-antibody complex was visualized with horseradish peroxidase (HRP)-conjugated secondary antibodies (P447 or P448, diluted 1:3000, Dako) using the ECL system (Amersham Pharmacia Biotech) or visualized with LI-COR IRDye-conjugated secondary antibodies (IRDye 926-32213 and 926-32212 in the 700 and 800 nm channel, diluted 1:12000, LI-COR Biosciences, Cambridge, UK). Odyssey Infrared Imager (LI-COR Biosciences, Cambridge, UK) connected to a computer running Odyssey v. 12 was used for visualization. ECL films were digitalized using an Epson Perfection 2450 scanner and all band densities were quantified by using ImageJ [28]. The specific bands were corrected to the Coomassie gels and normalized to the mean of control bands.

### Primary antibodies

For semiquantitative immunoblotting, we used previously characterized monoclonal and polyclonal antibodies as summarized below.

AQP2 (H7661), an affinity-purified rabbit polyclonal antibody raised against AQP2 has previously been described [29].

pS256-AQP2 (KO307), an affinity-purified rabbit polyclonal antibody raised against pS256-AQP2 has previously been described [30,31].

V2R (7251 AP): an affinity-purified rabbit polyclonal antibody raised against V2R has previously been described [32].

AQP3 (1591AP): an affinity-purified rabbit polyclonal antibody raised against AQP3 has previously been described [33,34].

AQP1 (2353 AP fr. 2–5): an affinity-purified rabbit polyclonal antibody raised against AQP1 has previously been described [35].

Na-K-ATPase α1-subunit 3B: an affinity-purified mouse monoclonal antibody raised against the Na-K-ATPase α1-subunit has previously been described [26,36].

The following commercial antibodies used in this study are summarized below.

AQP4 GST fusion peptide (Alomone labs, Jerusalem, Israel)

Heteromeric G protein subunit Gsα 371732 (Calbiochem-Novabiochem, San Diego, CA)

AT1 receptor (sc-579 rabbit polyclonal, Santa Cruz Biotechnology Inc.)

ATP6AP2 Renin receptor (ab40790, AbCam, Cambridge, UK)

### Grouping on gels

According to focus, all antibodies were incubated on gels containing the following combination of groups:

1. The regulation of early-stage HF on standard diet vs. enhanced RAS controls. Groups: Sham (n = 7); HF (n = 6); L-Sham (n = 8).
2. The regulation of early-stage HF with enhanced RAS vs. RAS enhanced controls. Groups: Sham (n = 7); L-HF (n = 7); L-Sham (n = 8).
3. The difference in regulation of standard diet HF, RAS enhanced HF, and RAS enhanced HF with DDAVP clamping. Groups: HF (n = 6); L-HF+d (n = 8); L-HF (n = 7).
4. The regulation of RAS enhanced HF and DDAVP clamping vs. RAS enhancement and DDAVP clamping in controls vs. RAS enhanced controls. Groups: L-Sham (n = 8); L-HF+d (n = 8); L-Sham+d (n = 8).
5. The regulation of RAS enhanced controls with DDAVP clamping vs. standard diet controls and RAS enhanced controls. Baseline conditions in healthy sham operated rats were thereby revealed during diet change and pharmacological interventions. Groups: Sham (n = 7); L-Sham+d (n = 8); L-Sham (n = 8).

### Statistical analysis

Data are expressed as means ± SE. Statistical significance between groups was estimated with one-way analysis of variance (ANOVA) followed by the Tukey-Kramer method for unequal sample sizes to test all possible pairwise differences of means to determine whether at least one difference was significantly different from 0. When assumptions for ANOVA were not fulfilled, Kruskal-Wallis nonparametric test was performed. *P* values < 0.05 were considered significant.

## Results

### Echocardiographic analysis

Data are presented in Tables 1 and 2. Table 1 displays the baseline echocardiographic findings day ten, retrieved before the allocation of the supplemental subgroups. At baseline all echocardiographic parameters changed in HF rats except heart rate with significant loss of cardiac function shown by ejection fraction (EF). EF was decreased from 70 ± 1% in Sham animals to 41 ± 1% in HF.

**Table 1:** Pretreatment echocardiagraphic parameters.

	Sham	HF
n	34	34
EF, %	70 ± 1	41 ± 1*
HR, bpm	321 ± 4	315 ± 4

Values are expressed as means ± SE. These values were measured at day 10 in the experiment, i.e. 10 days after MI surgery. *n*, number of rats;

EF;

left ventricular ejection fraction HR, heart rate;

*P < 0.001 vs. Sham.

**Table 2:** Post treatment echocardiagraphic parameters.

	Sham	HF	L-sham	L-HF	L-sham+d	L-HF+d
n	11	11	11	11	12	12
EF, %	66 ± 1	42 ± 2*	72 ± 2* #	41 ± 3* ‡	70 ± 1* # ‡ ¤	41 ± 2* ‡ ^♣^
HR, bpm	326 ± 8	354 ± 5*	362± 7*	362 ± 6*	358 ± 4*	352 ± 8* ‡

Values are expressed as means ± SE. These values were measured at day 16 in the experiment, i.e. 16 days after MI surgery. *n*, number of rats;

EF; left ventricular ejection fraction;

HR, heart rate; bpm, beats per minute.

*P < 0.05 vs. Sham

# *P <* 0.05 vs. HF

‡ *P <* 0.05 vs. L-Sham

¤ *P <* 0.05 vs. L-HF

♣ *P <* 0.05 vs. L-Sham+d.

The rats were re-examined after another six days, shown in Table 2. All HF groups exhibited significant decreased EF and increased heart rate vs. Sham. No difference in EF among HF groups was observed. In contrast, L-Sham and L-Sham+d increased EF compared with Sham rats. These findings altogether indicated progression of cardiac disease not only in all HF groups but also in both sodium restricted sham groups.

### Clearance studies

Data are presented in Table 3. Mean body weight was unchanged among groups, and did not differ at any time in the study period (data not shown). Plasma osmolality was significantly decreased in all groups, including standard diet HF rats compared with sham rats 17 days after MI. L-HF developed the largest decline in plasma osmolality, whereas L-Sham+d and L-HF+d were comparable. Urine-to-plasma osmolality ratio increased in all sodium restricted groups except L-HF. Plasma urea was significantly increased in L-HF vs. L-Sham, and in L-HF+d vs. L-Sham+d. Urine urea increased in L-Sham, L-Sham+d, and L-HF+d, respectively. Electrolyte free water reabsorption takes the amount of urea in urine and plasma into account. Electrolyte free water reabsorption (T^e^cH_2_O) was expectedly decreased in all sodium restricted groups, but was slightly increased in the two DDAVP groups vs. L-Sham. Consistently, DDAVP groups decreased urine output on the last day of monitoring compared with the other sodium restricted groups, despite of comparable water intake. HF rats exhibited decreased levels of fractional urinary excretion of sodium (FeNa) compared with sham rats in presence of unchanged creatinine clearance (Ccr), otherwise no difference among groups was observed. Urinary sodium excretion [urine sodium (UNa) x urine output (UO)] and urinary potassium excretion [urine sodium (UK) x urine output (UO)] were decreased in all sodium restricted groups compared with Sham and HF rats. Plasma sodium was significantly decreased in HF rats compared with Sham rats, and in L-HF and L-HF+d rats vs. L-Sham. Plasma potassium remained within standard range among groups. Substantial decreases in Ccr and plasma creatinine were observed in L-HF and L-HF+d compared with all control groups. Together with hyponatremia these results are consistent with cardiac decompensation.

**Table 3:** Changes in renal function.

	Sham	HF	L-sham	L-HF	L-sham+d	L-HF+d
n	11	11	11	11	12	12
BW, g	363 ± 8	358 ± 7	360 ± 12	358 ± 6	354 ± 8	360 ± 6
Water intake, ml	32 ± 1	32 ± 1	18 ± 1* #	22 ± 1* #‡	17 ± 1* #	16 ± 1* #
UO, μl·min^-1^ ·kg^-1^	21.4 ± 1.8	24.5 ± 1.8	13.0 ± 1.3* #	15.5 ± 2.0* #	8.3 ± 0.9* # ‡ ¤	8.2 ± 1.2* # ‡ ¤
U-Osm, mosm/KgH_2_O	2217 ± 113	2134 ± 131	2444 ± 180	2171 ± 183	3279 ±73* # ‡ ¤	3273 ± 101*#‡ ¤
P-Osm, mosm/KgH_2_O	299 ± 1	296 ± 1*	287 ± 7*	245 ± 15*#‡	281 ± 4*#¤	284 ± 4* # ¤
U/P-Osm	7.4 ± 0.4	7.4 ± 0.4	9.6 ± 0.6* #	7.6 ± 0.4# ‡	11.3 ± 0.2* # ‡ ¤	11.1 ± 0.4* # ‡ ¤
P-Urea, mmol/l	7.9 ± 0.4	7.9 ± 0.2	6.4 ± 0.5* #	7.7 ± 0.3‡	7.5 ± 0.2‡	8.2 ± 0.2‡ ^♣^
U-Urea, mmol/l	1219 ± 58	1320 ± 164	1731 ± 155* #	1371 ± 168	2477 ± 111* # ‡ ¤	2394 ± 114* # ‡ ¤
T^e^cH_2_O, μl/min	24.8 ± 3.3	23.0 ± 1.9	5.5 ± 0.6* #	6.2 ± 0.7* #	6.8 ± 1.0* # ‡	7.0 ± 0.9* # ‡
FeNa, %	0.643 ± 0.040	0.443 ± 0.037*	0.009 ± 0.002* #	0.010 ± 0.001* #	0.013 ± 0.003* #	0.010 ± 0.001* #
UNa x UO, mmol	1.38 ± 0.19	1.36 ± 0.11	0.03 ± 0.00* #	0.03 ± 0.01* #	0.04 ± 0.01* #	0.02 ± 0.00* #
UK x UO, mmol	4.9 ± 0.5	4.9 ± 0.3	2.0 ± 0.2* #	2.3 ± 0.2* # ‡	1.9 ± 0.2* #	2.0 ± 0.3* #
P-Na, mmol/l	139 ± 1	136 ± 0*	137 ± 0*	134 ± 1* ‡	136 ± 0*	130 ± 4* ‡ ^♣^
P-K, mmol/l	5.0 ± 0.1	5.0 ± 0.1	4.8 ± 0.1* #	4.5 ± 0.2* #	4.3 ± 0.1* # ‡	4.5 ± 0.1* #
P-Cr, μmol/l	44 ± 2	40 ± 1	36 ± 2* #	41 ± 1‡	38 ± 1*	37 ± 1* ¤
Ccr, ml/min	1.49 ± 0.08	1.50 ± 0.07	1.81 ± 0.17	1.18 ± 0.07* # ‡	1.39 ± 0.05‡ ¤	1.17 ± 0.09* # ‡ ^♣^

Values are expressed as means ± SE. The plasma values are measured at the last day of experiment whereas urine values and body weights are measured the day before to avoid error due to anesthesia under echocardiographic measurements. n, number of rats;

BW, median body weight;

Water intake, water intake;

UO, urine output; U-Osm, urine osmolality;

P-Osm, plasma osmolality;

U/P Osm, urine-to-plasma osmolality ratio;

P-Urea, plasma urea;

U-Urea, urine urea;

T^e^cH_2_O, electrolyte free water reabsorption;

FENa, fractional excretion of sodium into urine;

UNa x UO, rate of urinary sodium excretion;

UK x UO, rate of urinary potassium excretion;

P-Na, plasma sodium;

P-K, plasma potassium;

P-Cr, plasma creatinine;

Ccr, creatinine clearance.

*P < 0.05 vs. Sham

# *P <* 0.05 vs. HF

‡ *P <* 0.05 vs. L-Sham

¤ *P <* 0.05 vs. L-HF

♣ *P <* 0.05 vs. L-Sham+d.

### Blunted increase of AQP2 and p(Ser256)-AQP2 abundance in all HF groups

**Fig 2:**
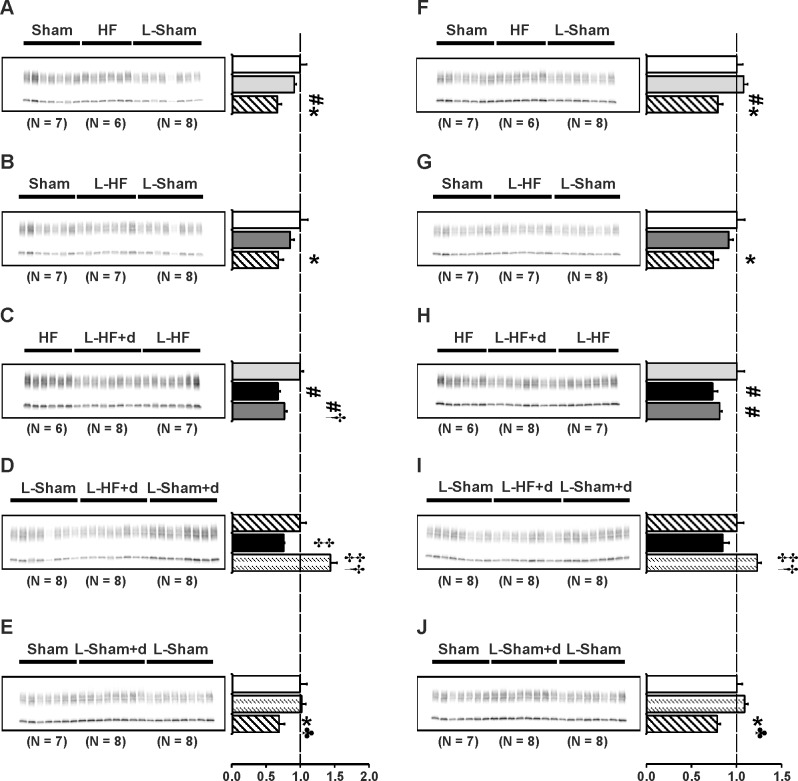
AQP2 and p-AQP2 abundance. Semiquantitative immunoblotting of kidney protein prepared from inner medulla. Immunoblot was reacted with anti-AQP2 (*A-E*) and AQP2 phosphorylated at Ser256 (p-AQP2) (*F-J*) antibody. Both antibodies reveal specific 29 kDa and 35–50 kDa bands. Data are presented in Table 4. Densitometric analysis revealed unchanged AQP2 and p-AQP2 protein levels in HF and L-HF vs. Sham 17 days after MI. Neither sodium restriction nor DDAVP infusion increased AQP2 and p-AQP2 abundance in HF as otherwise observed in L-Sham+d (*A*, *B* and *F*, *G*). Consistently, L-HF+d revealed decreased AQP2 and p-AQP2 abundance to L-HF levels vs. L-Sham and HF (*C*, *D* and *H*, *I*). Furthermore, AQP2 and p-AQP2 expression was decreased in L-Sham rats compared with Sham, HF, and L-Sham+d (*A*, *B*, *E* and *F*, *G*, *J*, respectively). In contrast, no difference was observed in L-Sham+d was observed vs. Sham (*E* and *J*). Each column represents the mean ± SE. Each column represents the mean ± SE. Solid white, Sham; solid light grey, HF; line pattern, L-Sham; solid dark grey, L-HF; solid black, L-HF+d; dotted pattern, L-Sham+d. *P < 0.05 vs. Sham, # *P <* 0.05 vs. HF, † *P <* 0.05 vs. L-HF+d, ‡ *P <* 0.05 vs. L-Sham, ♣ *P <* 0.05 vs. L-Sham+d.

Previously, we demonstrated that chronic HF 21 and 29 days after MI was associated with increased protein levels of AQP2 and p-AQP2 in IM. Semiquantitative immunoblotting was carried out to examine IM kidney abundance of AQP2 and p-AQP2. The purposes were: 1) to test whether changes in expression of AQPs play an important role in the early stages of HF before cardiac remodeling is complete. 2) to test whether the changes in plasma sodium and osmolality could be, in part, explained by changes in IM aquaporin abundances. Immunoblots are presented in Fig. 2 and the corresponding data in Table 4. In contrast to our previous findings in HF rats 21 and 29 days after MI [9,11], there was no change in AQP2 and p-AQP2 protein levels when compared between Sham groups and HF groups 17 days after MI. Neither sodium restriction nor DDAVP infusion increased AQP2 and p-AQP2 abundance in HF (Fig. 2, A, B and F, G). Indeed, the two sodium restricted HF groups revealed decreased AQP2 and p-AQP2 abundances vs. HF (Fig. 2, C and H). In contrast, AQP2 and p-AQP2 abundances were increased in L-Sham+d vs. L-Sham, as previously described (Fig. 2, D, E and I, J) [8].

**Table 4:** Inner medullary expression of AQP2 and p-AQP2.

AQP2			
A	Sham	HF	L-Sham
n	7	6	8
Fraction of Sham	100 ± 10	91 ± 3	66 ± 6*#
B	Sham	L-HF	L-Sham
n	7	7	8
Fraction of Sham	100 ± 11	85 ± 6	68 ± 7*
C	HF	L-HF+d	L-HF
n	6	8	7
Fraction of HF	100 ± 5	68 ± 3# †	78 ± 4#
D	L-Sham	L-HF+d	L-Sham+d
n	8	8	8
Fraction of L-Sham	100 ± 9	76 ± 1‡	145 ± 10‡ †
E	Sham	L-Sham+d	L-Sham
n	7	8	8
Fraction of Sham	100 ± 10	103 ± 6	69 ± 8* ^♣^
p-AQP2			
F	Sham	HF	L-Sham
n	7	6	8
Fraction of Sham	100 ± 7	108 ± 4	79 ± 5* #
G	Sham	L-HF	L-Sham
n	7	7	8
Fraction of Sham	100 ± 9	91 ± 5	74 ± 6*
H	HF	L-HF+d	L-HF
n	6	8	7
Fraction of HF	100 ± 8	73 ± 6#	81 ± 3#
I	L-Sham	L-HF+d	L-Sham+d
n	8	8	8
Fraction of L-Sham	100 ± 8	84 ± 7	122 ± 5‡ †
J	Sham	L-Sham+d	L-Sham
n	7	8	8
Fraction of Sham	100 ± 6	109 ± 4	78 ± 4* ^♣^

Values are expressed as means ± SE. AQP2, aquaporin-2;

p-AQP2, p(Ser256)-aquaporin-2;

n, number of rats.

*P < 0.05 vs. Sham

# *P <* 0.05 vs. HF

† *P <* 0.05 vs. L-HF+d

‡ *P <* 0.05 vs. L-Sham

♣ *P <* 0.05 vs. L-Sham+d. Values are expressed as means ± SE.

Phosphorylation at the Ser256 site near the COOH terminal of the AQP2 molecule is necessary for hormone regulated shuttling of AQP2 to the apical plasma membrane [30,37]. On this background we wanted to test, whether the observed changes in total AQP2 were accompanied by similar changes in p-AQP2. The changes of IM p-AQP2 in Fig. 2, F-J were comparable with the changes observed in AQP2, except there were no statistically changes in p-AQP2 abundances between the L-HF+d and L-HF rats (Fig. 2, C and H).

### AQP2 and p(Ser256)-AQP2 distribution remained unchanged in all HF groups

**Fig 3:**
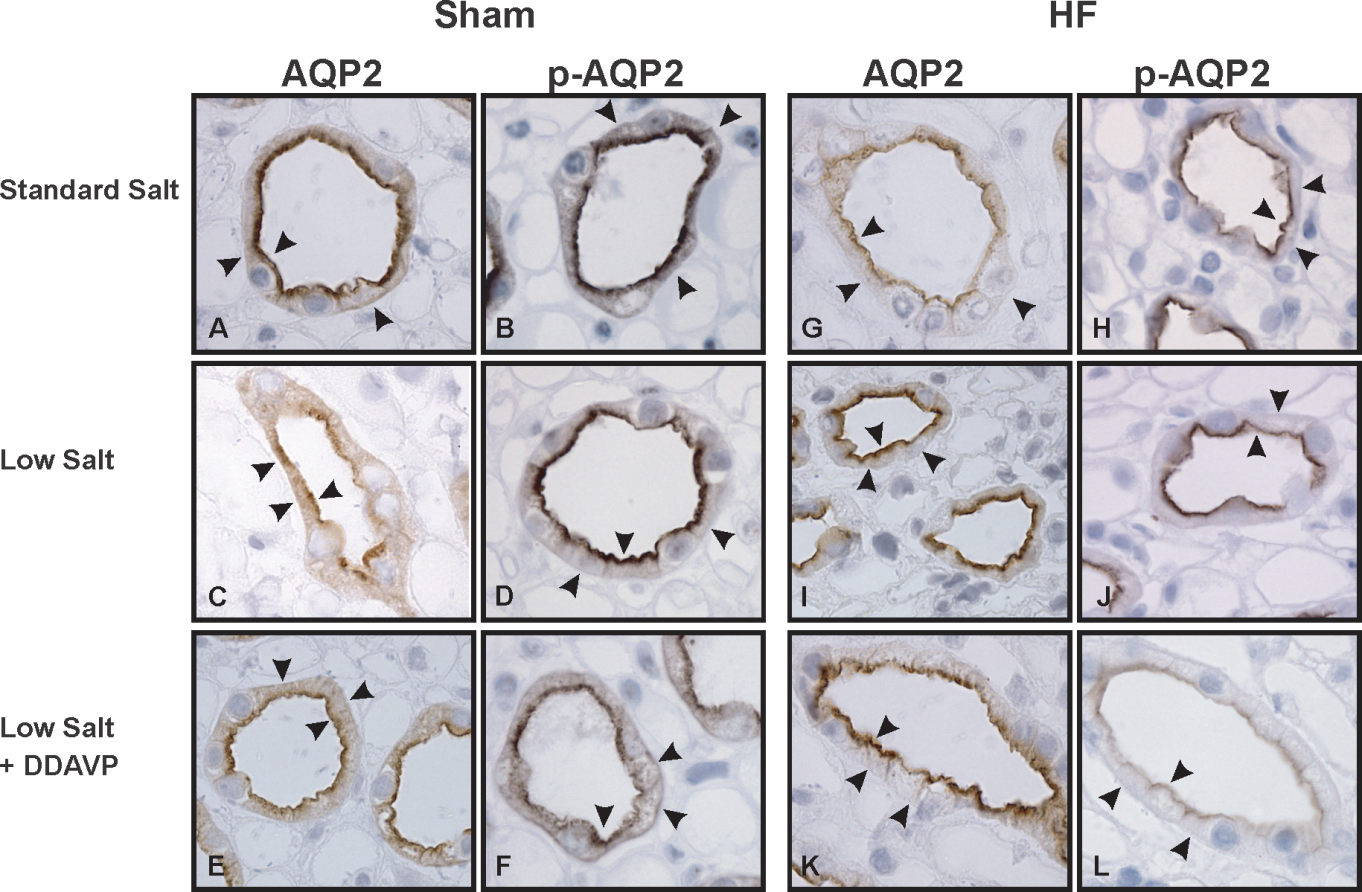
AQP2 and p-AQP2 IMCD localization. Immunoperoxidase microscopy of AQP2 and p-AQP2 in the inner medulla. Sections were incubated with affinity-purified anti-AQP2 (*A – E*) or affinity-purified anti-p-AQP2 antibody (*F* – *J*), and labeling was visualized using peroxidase-conjugated secondary antibody. *A – B*: In standard salt diet Sham rats AQP2 and p-AQP2 labeling is present at the apical and intracellular domains (arrows). *C – F*: AQP2 and p-AQP2 stainings from L-Sham and L-Sham+d rats were mainly situated in apical domains with weak intracellular labeling. *G – L*: In contrast, all HF groups demonstrated strong apical immunoperoxidase labeling of AQP2 and p-AQP2 with virtually no staining in intracellular domains. Magnification x 630.

Additional immunocytochemistry was performed to investigate whether the observed changes in AQP2 and p-AQP2 by immunoblot were associated with changes in subcellular localization (Fig. 3, A – L). AQP2 and p-AQP2 demonstrated similar distribution and the findings were consistent with previous studies.

Intracellular and a minor amount of basal staining of AQP2 and p-AQP2 was observed in standard diet Sham from IMCD principal cells (Fig. 3, A and B) [38]. As shown previously, the AQP2 and p-AQP2 staining was observed mainly in the apical domains in HF rats (Fig. 3, G and H) [2,9,11]. L-Sham and L-Sham+d revealed similar distributions of AQP2 and p-AQP2 with mainly apical staining, (Fig. 3, C, D and E, F, respectively) [8]. In the HF groups a profound apical labeling with virtually no intracellular staining was observed. These changes in AQP2 and p-AQP2 distribution were similar between HF, L-HF, and L-HF+d, respectively (Fig. 3, G, H and I, J, and K, L).

### V2 vasopressin receptor abundance in inner medulla is only increased in HF rats and L-HF rats

**Fig 4:**
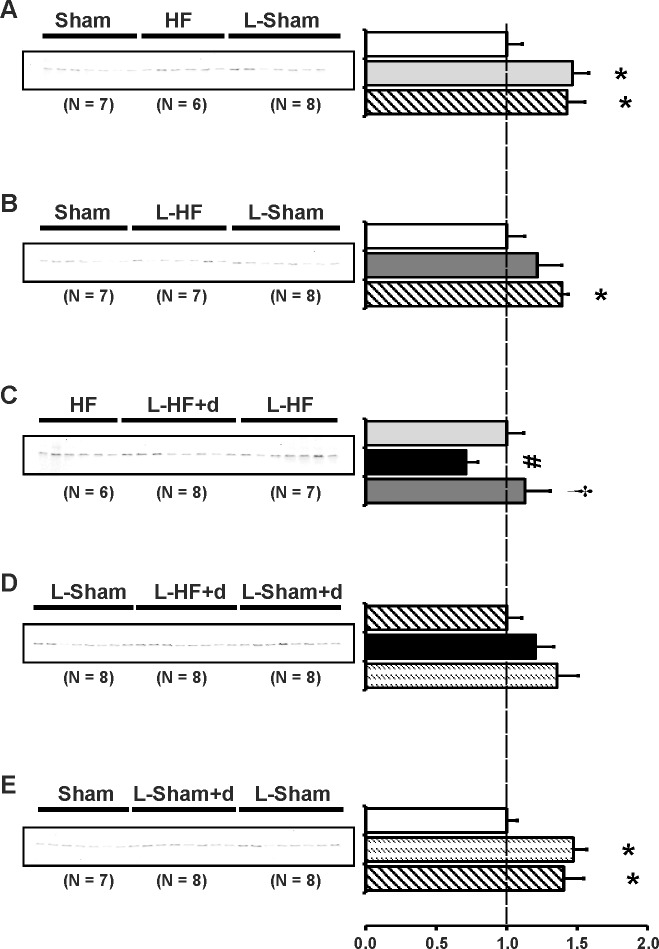
V2 vasopressin receptor abundance. Semiquantitative immunoblotting of kidney protein prepared from inner medulla. Immunoblot was reacted against anti-V2R protein and revealed a single band at ~ 47 kDa (*A-E*). Data are presented in Table 5. *A*) Densitometry revealed increased V2R expression in HF vs. Sham. A significant V2R increase was also present in L-Sham vs. Sham, as also seen in *B*) and *E*). *B*) The V2R expression between Sham and L-HF was comparable. *C*) Decreased V2R protein expression in L-HF+d vs. HF and L-HF was observed, whereas standard diet HF and L-HF was comparable. *D*) No statistical differences were found among groups. *E*) V2R abundance was increased in L-Sham+d and L-Sham vs. Sham, whereas L-Sham+d and L-Sham remained unchanged. Each column represents the mean ± SE. Solid white, Sham; solid light grey, HF; line pattern, L-Sham; solid dark grey, L-HF; solid black, L-HF+d; dotted pattern, L-Sham+d. *P < 0.05 vs. Sham, # *P <* 0.05 vs. HF, † *P <* 0.05 vs. L-HF+d.

To examine whether the observed changes in AQP2 and p-AQP2 could be due to changes in V2R abundance, semiquantitative immunoblotting was carried out. Immunoblots are presented in Fig. 4 and the corresponding data in Table 5. HF rats increased V2R to L-Sham levels vs. Sham (Fig. 4, A). In contrast, L-HF did not significantly increase vs. Sham and L-Sham (Fig. 4, B). No change between L-Sham, L-Sham+d, and L-HF+d was observed, but L-Sham and L-Sham+d increased V2R abundance vs. standard diet Sham (Fig. 4, D and E). In contrast, L-HF+d decreased V2R abundance vs. HF and L-HF rats (Fig. 4, C).

**Table 5:** Inner medullary expression of the V2 vasopressin receptor.

V2R			
A	Sham	HF	L-Sham
n	7	6	8
Fraction of Sham	100 ± 11	147 ± 12*	143 ± 13*
B	Sham	L-HF	L-Sham
n	7	7	8
Fraction of Sham	100 ± 13	122 ± 17	139 ± 5*
C	HF	L-HF+d	L-HF
n	6	8	7
Fraction of HF	100 ± 12	71 ± 8#	113 ± 18†
D	L-Sham	L-HF+d	L-Sham+d
n	8	8	8
Fraction of L-Sham	100 ± 11	120 ± 13	144 ± 15
E	Sham	L-Sham+d	L-Sham
n	7	8	8
Fraction of Sham	100 ± 7	147 ± 10*	140 ± 14*

Values are expressed as means ± SE. V2R, V2 vasopressin receptor;

n, number of rats.

*P < 0.05 vs. Sham

# *P <* 0.05 vs. HF

† *P <* 0.05 vs. L-HF+d.

### The Gsα subunit is increased in HF and L-HF rats but not in L-HF+d

**Fig 5:**
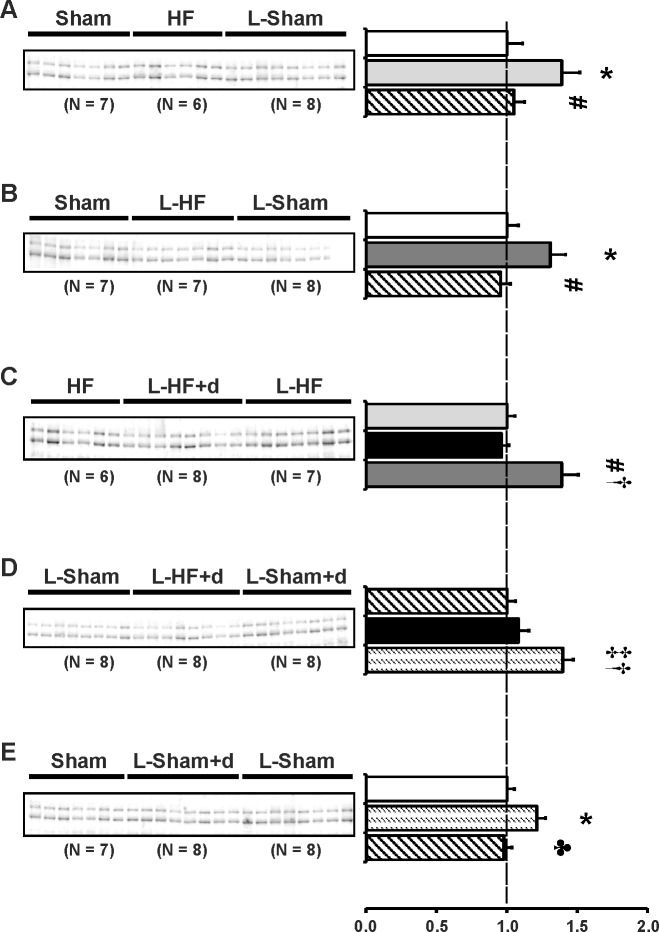
Gsα subunit abundance. Semiquantitative immunoblotting of kidney protein prepared from inner medulla. Immunoblot was reacted against anti-Gsα subunit of the G-protein revealing a doublet band at 45 and 50 kDa (*A-E*). Data are presented in Table 6. *A*) Densitometric analysis revealed significantly increased Gsα abundance in HF vs. Sham and L-Sham, whereas Gsα expression was comparable between Sham and L-Sham, as also presented in *B*) and *E*). *B*) Gsα was increased in L-HF vs. Sham and L-Sham. *C*) Gsα abundance was increased in L-HF rats vs. HF and L-HF+d, whereas Gsα expressions were comparable between HF and L-HF+d. *D*) Gsα abundance was increased in L-Sham+d rats vs. L-Sham rats and L-HF+d. L-Sham and L-HF+d groups were comparable. *E*) As observed in *D*), Gsα abundances in L-Sham+d rats were increased vs. Sham and L-Sham. Each column represents the mean ± SE. Solid white, Sham; solid light grey, HF; line pattern, L-Sham; solid dark grey, L-HF; solid black, L-HF+d; dotted pattern, L-Sham+d. *P < 0.05 vs. Sham, # *P <* 0.05 vs. HF, † *P <* 0.05 vs. L-HF+d, ‡ *P <* 0.05 vs. L-Sham, ♣ *P <* 0.05 vs. L-Sham+d.

To investigate whether the observed changes in V2R abundance were associated with concomitant changes in the associated protein G coupled pathway, semiquantitative immunoblotting was carried out. Immunoblots are presented in Fig. 5 and the corresponding data in Table 6. IM Gsα protein abundance was increased in HF and L-HF vs. Sham and L-Sham (Fig. 5, A and B). A similar increase was observed in L-Sham+d (Fig. 5, E). In contrast, L-HF+d remained comparable with L-Sham (Fig. 5, D).

**Table 6:** Inner medullary expression of the Gsα subunit.

Gsα			
A	Sham	HF	L-Sham
n	7	6	8
Fraction of Sham	100 ± 11	139 ± 13*	105 ± 8#
B	Sham	L-HF	L-Sham
n	7	7	8
Fraction of Sham	100 ± 8	131 ± 11*	95 ± 7#
C	HF	L-HF+d	L-HF
n	6	8	7
Fraction of HF	100 ± 6	96 ± 5	139 ± 12# †
D	L-Sham	L-HF+d	L-Sham+d
n	8	8	8
Fraction of L-Sham	100 ± 6	108 ± 8	140 ± 7† ‡
E	Sham	L-Sham+d	L-Sham
n	7	8	8
Fraction of Sham	100 ± 5	121 ± 6*	98 ± 6^♣^

Values are expressed as means ± SE. Gsα, Gsα subunit;

n, number of rats.

*P < 0.05 vs. Sham

# *P <* 0.05 vs. HF

† *P <* 0.05 vs. L-HF+d

‡ *P <* 0.05 vs. L-Sham

♣ *P <* 0.05 vs. L-Sham+d.

### The type-1 angiotensin II receptor is downregulated in HF and L-HF+d

**Fig 6:**
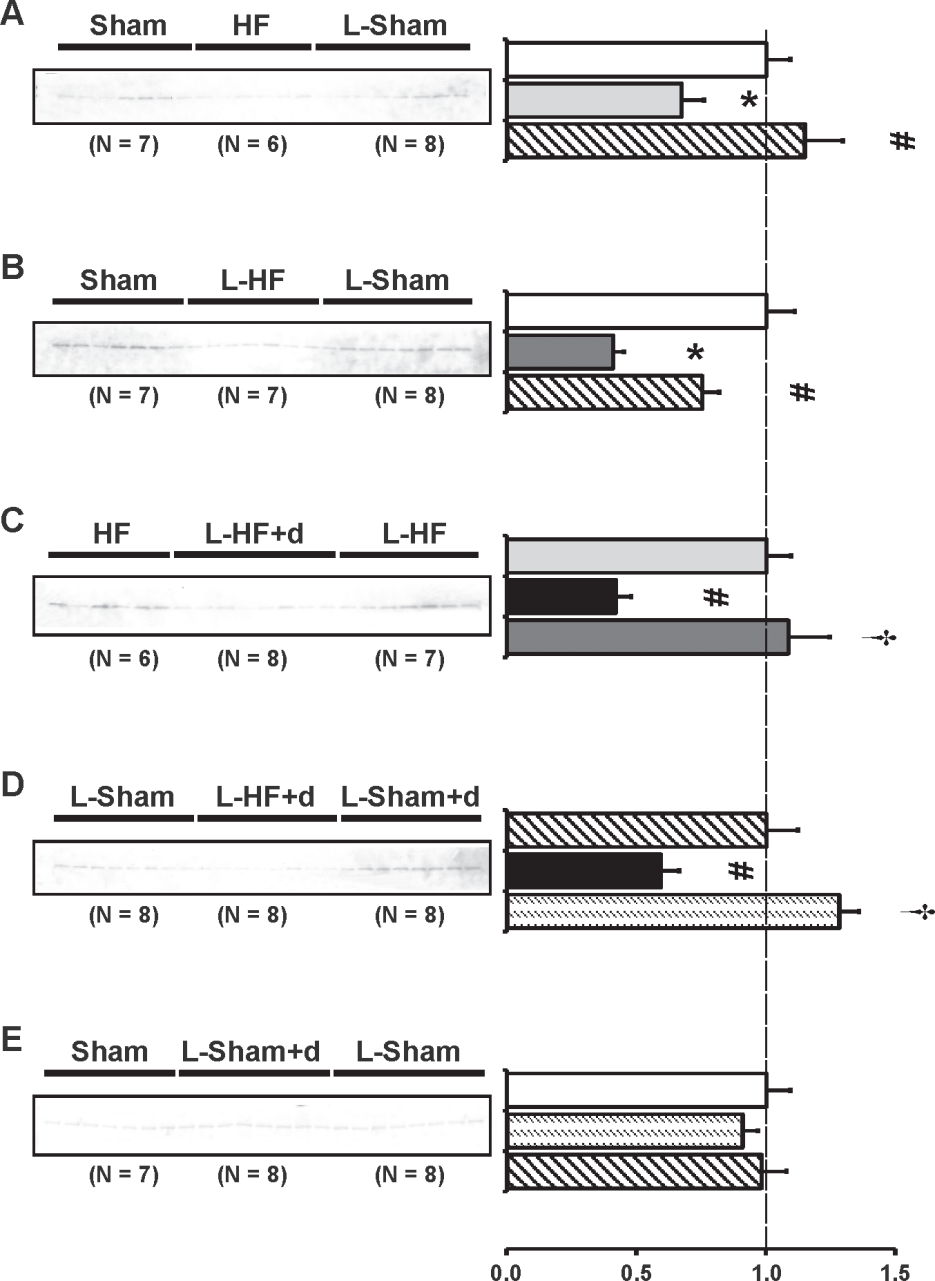
Type 1_A_ angiotensin II receptor abundance. Semiquantitative immunoblotting of kidney protein prepared from inner medulla. Immunoblot was reacted with anti-AT1R revealing a single band at ~ 43 kDa (*A-E*). Data are presented in Table 7. (*A* and *B*) Analysis revealed significantly decreased AT1R expression in HF and L-HF vs. Sham and vs. L-Sham, respectively. AT1R expression was comparable in Sham and L-Sham. *C*) When compared with HF and L-HF, AT1R protein expression was decreased in the L-HF+d rats, whereas levels between HF and L-HF were comparable. *D*) Decreased expression of AT1R was observed in L-HF+d rats when compared with L-Sham and L-Sham+d, whereas AT1R levels between L-Sham and L-Sham+d were comparable, as also presented in *E*). *E*) AT1R protein expression was comparable between Sham, L-Sham and L-Sham+d. Each column represents the mean ± SE. Solid white, Sham; solid light grey, HF; line pattern, L-Sham; solid dark grey, L-HF; solid black, L-HF+d; dotted pattern, L-Sham+d. *P < 0.05 vs. Sham, # *P <* 0.05 vs. HF, † *P <* 0.05 vs. L-HF+d.

Previous studies have indicated a possible crosstalk between the V2R and the AT1R, which could be important in the pathophysiology of early-stage HF 17 days after MI [8,9]. Low sodium diet with normal levels of potassium stimulates the endogenous production of ANG II whereas ALDO levels are kept near baseline [8]. Thus we examined whether the observed changes of AQP2 and p-AQP2 in the HF groups could be due to changes in intrarenal AT1R expression in IM. Immunoblots are presented in Fig. 6 and the corresponding data in Table 7. AT1R protein abundances were downregulated in HF and L-HF vs. both Sham and L-Sham (Fig. 6, A and B). Furthermore, AT1R from L-HF+d was decreased vs. HF and L-HF and vs. L-Sham and L-Sham+d (Fig. 6, C and D). No changes were observed between sham groups (Fig. 6, E).

**Table 7:** Inner medullary expression of the type 1_A_ angiotensin II receptor.

AT1R			
A	Sham	HF	L-Sham
n	7	6	8
Fraction of Sham	100 ± 9	67 ± 9*	115 ± 15#
B	Sham	L-HF	L-Sham
n	7	7	8
Fraction of Sham	100 ± 11	41 ± 4*	75 ± 6#
C	HF	L-HF+d	L-HF
n	6	8	7
Fraction of HF	100 ± 10	42 ± 6#	109 ± 16†
D	L-Sham	L-HF+d	L-Sham+d
n	8	8	8
Fraction of L-Sham	100 ± 12	59 ± 7#	128 ± 8†
E	Sham	L-Sham+d	L-Sham
n	7	8	8
Fraction of Sham	100 ± 9	91 ± 6	98 ± 10

Values are expressed as means ± SE. AT1R, type 1_A_ angiotensin II receptor;

n, number of rats.

*P < 0.05 vs. Sham

# *P <* 0.05 vs. HF

† *P <* 0.05 vs. L-HF+d.

### The (pro)renin receptor is upregulated in L-HF but not in L-Sham rats

**Fig 7:**
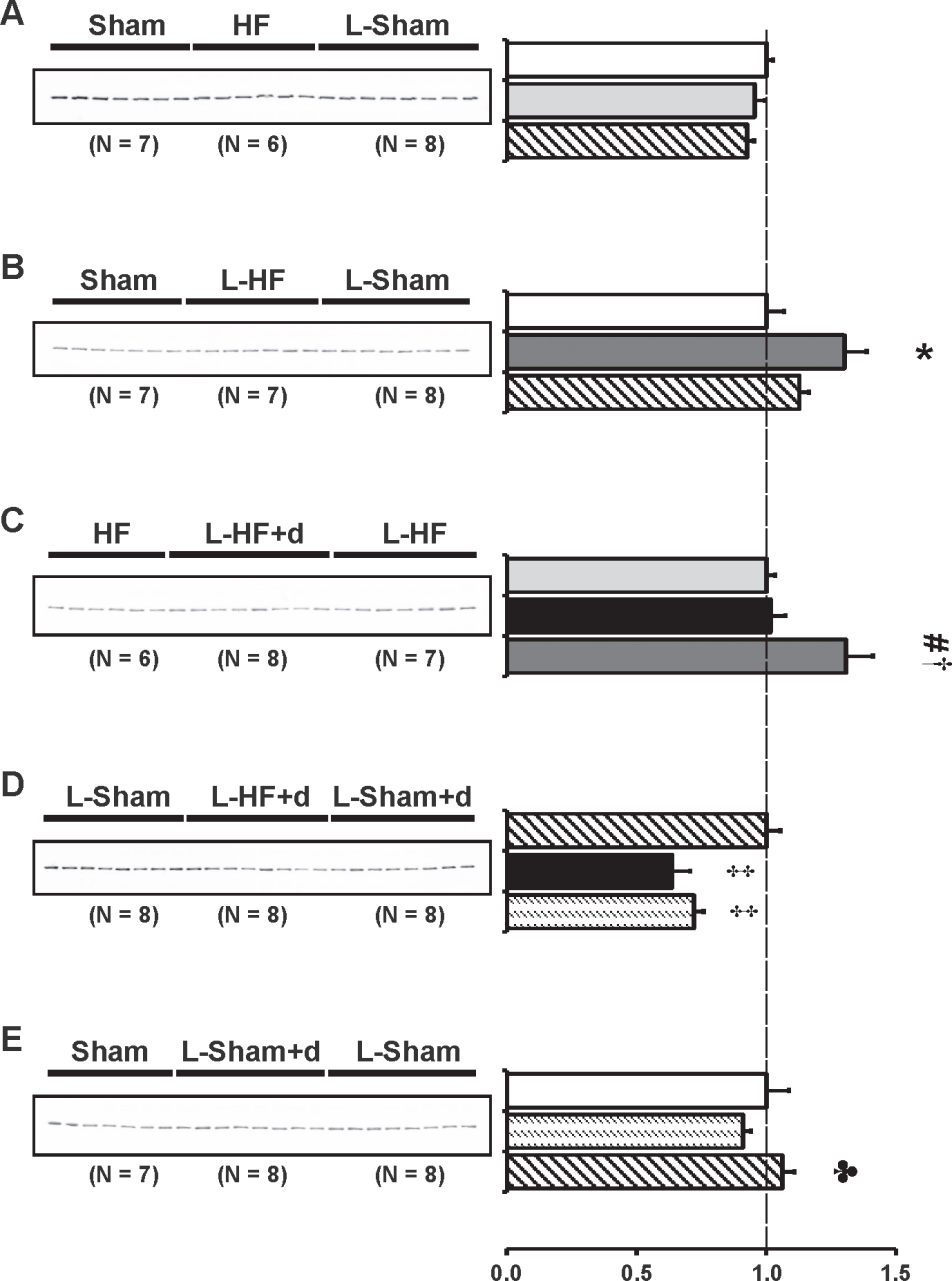
(Pro)renin receptor abundance. Semiquantitative immunoblotting of kidney protein prepared from inner medulla. Immunoblot was reacted with a specific antibody against anti-(P)RR revealing a single band at ~ 42 kDa (*A-E*). Data are presented in Table 8. (*A* and *B*) (P)RR expression was increased in L-HF vs. Sham and L-Sham, whereas no difference in (P)RR protein expression between Sham, HF or L-Sham was found. As also presented in *A*) and *E*), no difference was observed between Sham and L-Sham rats. *C*) (P)RR expression increased in L-HF vs. HF and L-HF+d, whereas no difference was observed between HF and L-HF+d. *D*) Densitometry revealed significantly decreased (P)RR abundance in L-HF+d and L-Sham+d when compared with L-Sham. The decrease in L-HF+d and L-Sham+d was comparable. *E*) As already shown, densitometry revealed that L-Sham+d decreased (P)RR expression compared with L-Sham, and no difference was found between Sham and L-Sham. Each column represents the mean ± SE. Solid white, Sham; solid light grey, HF; line pattern, L-Sham; solid dark grey, L-HF; solid black, L-HF+d; dotted pattern, L-Sham+d. *P < 0.05 vs. Sham, # *P <* 0.05 vs. HF, † *P <* 0.05 vs. L-HF+d, ‡ *P <* 0.05 vs. L-Sham, ♣ *P <* 0.05 vs. L-Sham+d.

The (pro)renin receptor ((P)RR) is expressed in intercalated type A cells in the collecting ducts (CD). In addition, soluble (P)RR is secreted into the tubular lumen. (P)RR has been shown to induce ANG I formation from angiotensinogen (AGT) from the proximal tubules (for review, see [39]). As the late CD’s contain angiotensin-converting enzyme facilitating the conversion of ANG I into ANG II which could modulate the regulation of water channels, we wanted to test whether changes in (P)RR abundance could play a role in the pathophysiology of early-stage HF, and whether this could be altered by RAS enhancement or clamped high-level DDAVP. Immunoblots are presented in Fig. 7 and the corresponding data in Table 8. No change in IM (P)RR was observed between Sham rats, L-Sham, and HF (Fig. 7, A). (P)RR was mildly increased in L-HF vs. Sham and the other HF groups but remained unchanged vs. L-Sham (Fig. 7, B and C). In contrast, L-Sham+d and L-HF+d decreased (P)RR levels vs. L-Sham. (Fig. 7, D).

**Table 8:** Inner medullary expression of the (pro)renin receptor.

(P)RR			
A	Sham	HF	L-Sham
n	7	6	8
Fraction of Sham	100 ± 2	95 ± 4	92 ± 3
B	Sham	L-HF	L-Sham
n	7	7	8
Fraction of Sham	100 ± 7	130 ± 9*	113 ± 4
C	HF	L-HF+d	L-HF
n	6	8	7
Fraction of HF	100 ± 3	102 ± 6	131 ± 10# †
D	L-Sham	L-HF+d	L-Sham+d
n	8	8	8
Fraction of L-Sham	100 ± 5	64 ± 7‡	72 ± 4‡
E	Sham	L-Sham+d	L-Sham
n	7	8	8
Fraction of Sham	100 ± 9	92 ± 3	106 ± 5^♣^

Values are expressed as means ± SE. (P)RR, (pro)renin receptor;

n, number of rats.

*P < 0.05 vs. Sham

# *P <* 0.05 vs. HF

† *P <* 0.05 vs. L-HF+d

‡ *P <* 0.05 vs. L-Sham

♣ *P <* 0.05 vs. L-Sham+d.

### Inner medullary expression of AQP3 and Na-K-ATPase

The abundance of the basolateral water channel AQP3 has previously been shown to remain unchanged in chronic stage HF rats [11]. AQP3 is partly regulated by AVP [40]. Thus, IM AQP3 could be altered in early-stage HF. Data are presented in Table 9. Semiquantitative immunoblotting showed that all groups maintained unchanged AQP3 protein levels, except L-HF+d which downregulated AQP3 expression compared with HF (Table 9, C) and L-Sham (Table 9, D), respectively. In addition, we investigated whether Na-K-ATPase protein expression was altered, as seen in previous studies [41], however no changes were observed (Table 9, A – E).

**Table 9:** Inner medullary expression of AQP3 and Na-K-ATPase.

A	Sham	HF	L-Sham
n	7	6	8
AQP3	100 ± 14	107 ± 18	99 ± 9
Na-K-ATPase	100 ± 31	67 ± 23	100 ± 20
B	Sham	L-HF	L-Sham
n	7	7	8
AQP3	100 ± 14	103 ± 16	93 ± 10
Na-K-ATPase	100 ± 26	87 ± 18	73 ± 17
C	HF	L-HF+d	L-HF
n	6	8	7
AQP3	100 ± 15	57 ± 11#	69 ± 11
Na-K-ATPase	100 ± 37	134 ± 27	121 ± 23
D	L-Sham	L-HF+d	L-Sham+d
n	8	8	8
AQP3	100 ± 8	59 ± 11‡	82 ± 10
Na-K-ATPase	100 ± 25	120 ± 26	98 ± 23
E	Sham	L-Sham+d	L-Sham
n	7	8	8
AQP3	100 ± 12	108 ± 10	93 ± 9
Na-K-ATPase	100 ± 29	103 ± 26	112 ± 25

Values are expressed as means ± SE. AQP3, aquaporin-3;

Na-K-ATPase, Na-K-ATPase;

n, number of rats.

# *P* < 0.05 vs. HF

‡ *P <* 0.05 vs. L-Sham.

### Inner medullary AQP1 abundance is decreased in low sodium diet Sham rats

**Fig 8:**
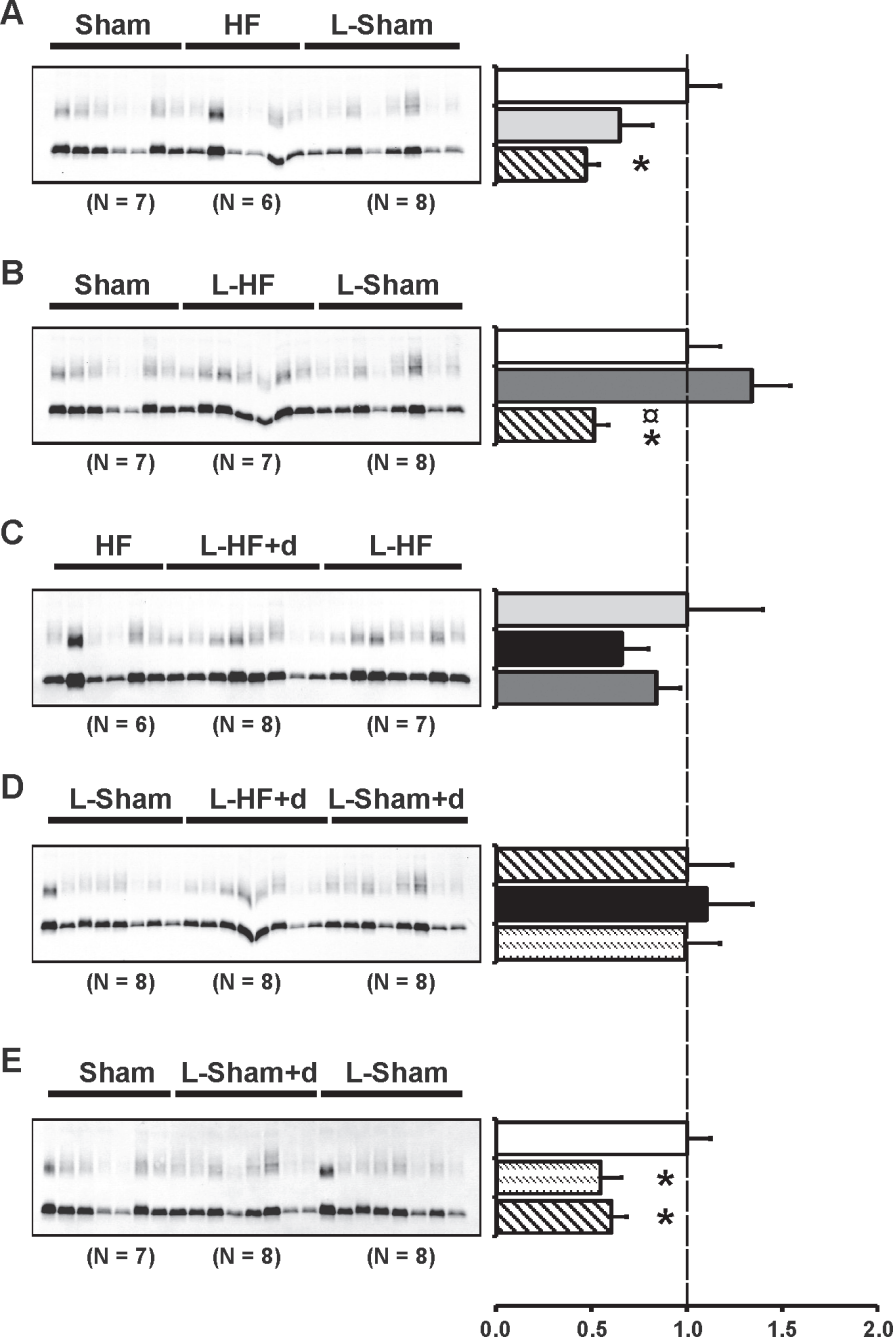
AQP1 abundance. Semiquantitative immunoblotting of kidney protein prepared from inner medulla. Immunoblot was reacted with anti-AQP1 antibody and reveals 29 kDa and 35–50 kDa AQP1-bands (*A-E*). Data are presented in Table 9. *A*) L-Sham was decreased vs. Sham and HF, as also presented in *B*) and *E*). No difference was found between Sham and HF. *B*) No difference was observed between Sham and L-HF. *C*) HF, L-HF+d, and L-HF had comparable AQP1 protein levels. *D*) Densitometric analysis revealed that L-Sham, L-HF+d, and L-Sham+d had comparable AQP1 protein levels. Each column represents the mean ± SE. Solid white, Sham; solid light grey, HF; line pattern, L-Sham; solid dark grey, L-HF; solid black, L-HF+d; dotted pattern, L-Sham+d. *P < 0.05 vs. Sham, ¤ *P <* 0.05 vs. L-HF.

AQP1 is constitutively expressed in IM thin descending limps origin from long loop nephrons [42]. AQP1 is critical for urine concentration ability. Thus, we wanted to test AQP1 protein abundance in response to low sodium diet and DDAVP in early-stage HF, as these factors could alter IM tonicity [43]. Immunoblots are presented in Fig. 8 and the corresponding data in Table 10. AQP1 abundance remained unaffected in all the HF-groups, also when compared to Sham (Fig. 8, A,B, and C). In contrast, AQP1 was downregulated in L-Sham and L-Sham+d vs. Sham (Fig. 8, E).

**Table 10:** Inner medullary expression of AQP1.

AQP1			
A	Sham	HF	L-Sham
n	7	6	8
Fraction of Sham	100 ± 17	65 ± 17	47 ± 7*
B	Sham	L-HF	L-Sham
n	7	7	8
Fraction of Sham	100 ± 17	134 ± 20	51 ± 7* ¤
C	HF	L-HF+d	L-HF
n	6	8	7
Fraction of HF	100 ± 40	66 ± 14	84 ± 13
D	L-Sham	L-HF+d	L-Sham+d
n	8	8	8
Fraction of L-Sham	100 ± 24	110 ± 24	99 ± 18
E	Sham	L-Sham+d	L-Sham
n	7	8	8
Fraction of Sham	100 ± 12	55 ± 11*	60 ± 8*

Values are expressed as means ± SE. AQP1, aquaporin-1;

n, number of rats. L-Sham+d.

*P < 0.05 vs. Sham

¤ *P <* 0.05 vs. L-HF.

### Inner medullary AQP4 is increased in HF and L-HF rats

**Fig 9:**
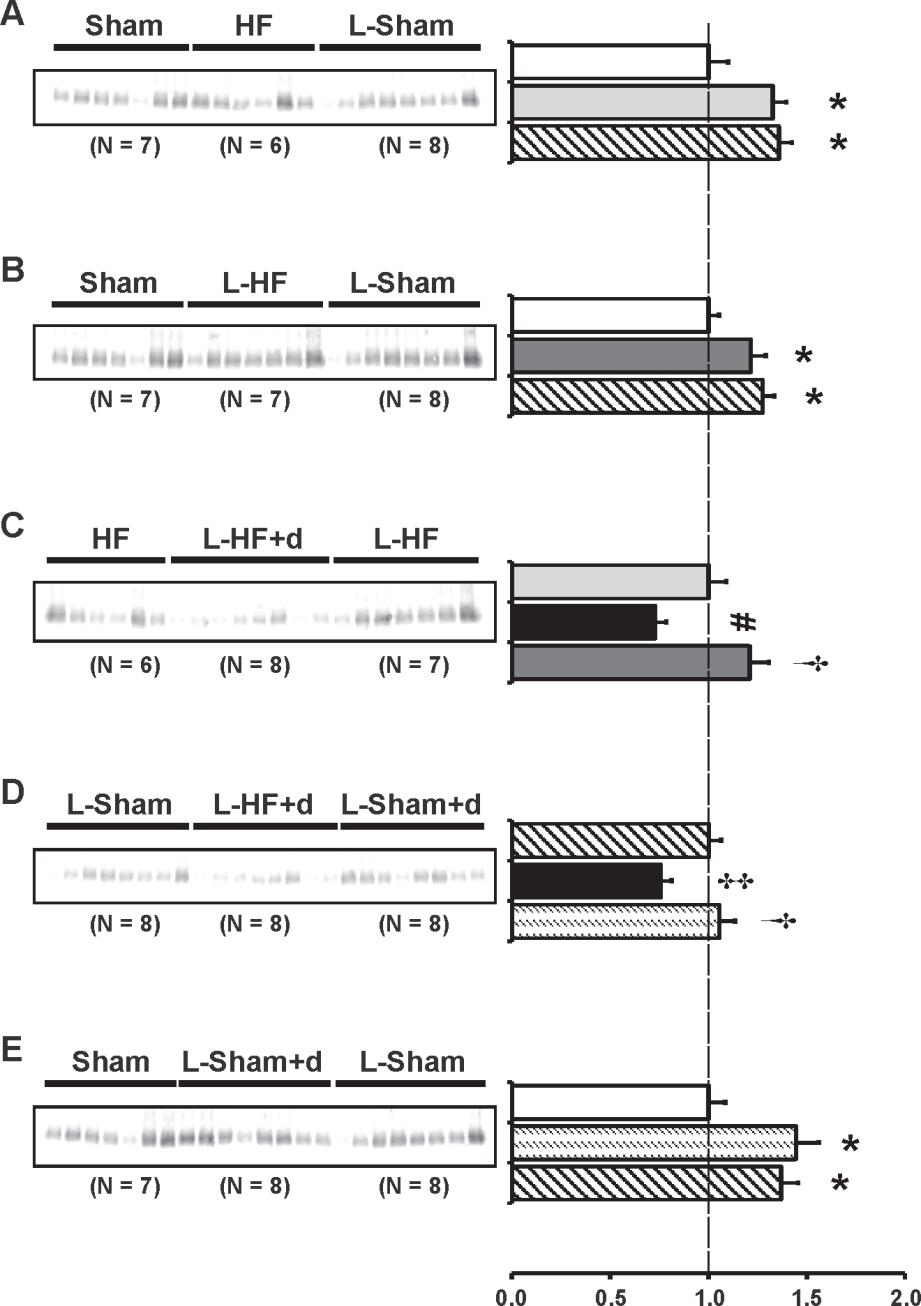
AQP4 abundance. Semiquantitative immunoblotting of kidney protein prepared from inner medulla. Immunoblot was reacted with anti-AQP4 antibody and reveals a single ~ 34.5 kDa AQP4-band (*A-E*). Data are presented in Table 10. (*A-B*) Densitometric analysis revealed significantly increased AQP4 protein levels of HF, L-HF and L-Sham vs. Sham. The increase in HF, L-HF and L-Sham was comparable. In contrast, AQP4 protein levels in L-HF+d decreased vs. HF and L-HF in *C*) and vs. L-Sham and L-Sham+d in *D*). No difference was observed between L-Sham and L-Sham+d, as also presented in *E*). *E*) AQP4 protein levels in L-Sham+d and L-Sham were increased compared with Sham. No difference was observed between L-Sham and L-Sham+d. Each column represents the mean ± SE. Solid white, Sham; solid light grey, HF; line pattern, L-Sham; solid dark grey, L-HF; solid black, L-HF+d; dotted pattern, L-Sham+d. *P < 0.05 vs. Sham, #*P <* 0.05 vs. HF, † *P <* 0.05 vs. L-HF+d, ‡ *P <* 0.05 vs. L-Sham.

AQP4 is present at the basolateral membrane on IM principal cells where it in conjunction with AQP3 is facilitating water transport to the intracellular space [44]. Thus, we tested whether AQP4 could play a role in the fine tuning of IM free water reabsorption in HF and in settings where ANG II and V2R stimulation is increased. Immunoblots are presented in Fig. 9 and the corresponding data in Table 11. AQP4 abundance increased in HF, and L-HF, L-sham, and L-Sham+d vs. Sham (Fig. 9, A, B, and E). In contrast, L-HF+d downregulated AQP4 vs. HF, L-HF, L-Sham, and L-Sham+d (Fig. 9, C and D).

**Table 11:** Inner medullary expression of AQP4.

AQP4			
A	Sham	HF	L-Sham
n	7	6	8
Fraction of Sham	100 ± 10	133 ± 7*	136 ± 7*
B	Sham	L-HF	L-Sham
n	7	7	8
Fraction of Sham	100 ± 5	121 ± 8*	128 ± 6*
C	HF	L-HF+d	L-HF
n	6	8	7
Fraction of HF	100 ± 9	73 ± 6#	121 ± 10†
D	L-Sham	L-HF+d	L-Sham+d
n	8	8	8
Fraction of L-Sham	100 ± 6	76 ± 5‡	105 ± 8†
E	Sham	L-Sham+d	L-Sham
n	7	8	8
Fraction of Sham	100 ± 9	144 ± 12*	137 ± 9*

Values are expressed as means ± SE. AQP4, aquaporin-4;

n, number of rats.

*P < 0.05 vs. Sham

# *P <* 0.05 vs. HF

† *P <* 0.05 vs. L-HF+d

‡ *P <* 0.05 vs. L-Sham.

## Discussion

HF rats developed hyponatremia, hypo-osmolality, increased HR, and decreased levels of fractional urinary excretion of sodium, but exhibited comparable IM AQP2 and p-AQP2 abundance with Sham rats 17 days after MI. In previous HF studies 21, 29 and 34 days after MI, upregulation of AQP2 and distribution of AQP2 and p-AQP2 to apical domains in IMCD was observed [2,9,11,45]. Here we demonstrate that in early-stage HF, IMCD AQP2 and p-AQP2 are located in apical domains whereas AQP2 protein abundance remains comparable with Sham rats. This strongly indicates for the first time that the transit to chronically elevated AQP2 levels in HF happens in coordination with the finalization of myocardial remodeling.

### HF groups and sodium restricted sham rats had comparable decrements in plasma sodium and osmolality

All HF groups were hyponatremic with decreased plasma osmolality, disregarded the type of diet. HF rats on standard diet developed milder hyponatremia than the other groups. We used dietary sodium depletion and DDAVP administration to further enhance the effect of circulating ANG II and AVP. In previous studies using the same HF model, the HF rats had increased plasma levels of renin, ANG II, aldosterone, and AVP, in addition to increased abundance and apical targeting of AQP2, even when normonatremic and disregarding changes in plasma osmolality [2,9,46]. Hence, our present findings suggest that even in early-stage HF, elevated ANG II and AVP can mediate avid water retention. Sodium restricted sham groups lowered plasma sodium and osmolality to levels comparable with the HF groups. It is however surprising, that the L-HF+d and L-Sham+d rats displayed increased plasma osmolality compared with L-HF, despite of reduced urine output and comparable water intake, because plasma urea was also unchanged. We cannot fully explain this finding. However, on the last day of experiment, urine output in the L-HF+d rats was as low as 8.2 ± 1.2 μl·min^-1^ ·kg^-1^, almost half the output of the L-HF group. Therefore, a degree of uremia which in part can account for the increased plasma osmolality in the L-HF+d rats group cannot be ruled out. Nevertheless, the HF groups in our study managed to maintain AQP2 and p-AQP2 abundance within sham levels under physiological and hormonal conditions well-known to otherwise promote AQP2 abundance, suggesting activation of a compensating mechanism in early-stage HF not previously described [47,48].

### Vasopressin-induced AQP2 and p-AQP2 upregulation was blunted in early-stage HF after MI

Previous studies have shown that sodium restricted rats subjected to long-term DDAVP administration increased IM AQP2 and p-AQP2 [8]. We observed the same response in the L-Sham+d rats in our study. In the study by Kwon et al., AQP2 and p-AQP2 abundance was restored to control levels by co-treatment with the specific AT1R blocker candesartan [8]. Likewise, chronic-stage HF rats with elevated plasma ANG II levels reversed the observed increased protein expression and apical targeting of AQP2 and p-AQP2 in the IMCD after candesartan treatment [9]. In the present study, HF groups decreased AT1R protein abundance but elevated V2R and Gsα protein, indicating activation of the 3'-5'-cyclic adenosine monophosphate (cAMP)/protein kinase A (PKA) pathway [10]. AT1R is situated throughout the entire nephron including renal interstitial cells and vasculature to allow regulation of glomerular filtration rate (GFR), renal blood flow, and water and salt reabsorption [49]. Thus, there are strong indications that crosstalk between the AVP and ANG II signaling pathways is possible and important in HF. However, the underlying mechanism in V2R-AT1R crosstalk is still debated.

AVP-binding to V2R increases cAMP levels and mobilizes intracellular [Ca^2+^] stores in IMCD which in turn promotes AQP2-trafficking to the apical plasma membrane [50–53]. It has been suggested that Ca^2+^ is necessary for the insertion of AQP2 into the apical plasma membrane through calmodulin-dependent release from ryanodine-sensitive intracellular Ca^2+^ stores [54,55]. Furthermore, AQP2 trafficking can also be modulated through V2R regulated Ca^2+^ flux without affecting [cAMP] and through activation of the cAMP sensor protein Epac (exchange protein directly activated by cAMP) [10,56,57].

In general, ANG II induces a rise in intracellular [Ca^2+^] by inositol 1,4,5-triphosphate [58] and PKC activation by PLC mediated formation of the second messenger diacylglycerol (DAG) [59]. ANG II also has the capability to induce a large variety of additional intracellular signal cascades involving AQP2 regulation, including phosphorylated mitogen-activated protein (MAP) kinase, extracellular signal–regulated kinase 1/2 (ERK1/2), p38 kinase, and phosphatidylinositol 3'-kinase [60]. Evidence of PKC mediated cAMP accumulation has been shown in transfected HEK-293 cells. More direct potentiation of cAMP accumulation by ANG II through activation of protein kinase C (PKC) and intracellular Ca^2+^ release has also been shown. Here, ANG II enhanced DDAVP-mediated AQP2 targeting in V2R and AT1R transfected Chinese hamster ovary cells and in primary cultured IMCD cells [10,55]. Since rise in intracellular [Ca^2+^] induced by thapsigargin did not enhance cAMP accumulation when adding AVP, it was concluded that Ca^2+^ flux is less likely to be the prime mechanism of AT1R-V2R crosstalk [10]. However, some inconsistency exists in the current literature. For example, thapsigargin increased intracellular [Ca^2+^] in rat cardiac fibroblasts and potentiated isoproterenol- and forskolin-stimulated cAMP production. This study suggests that ANG II mediated potentiation of cAMP is facilitated by phospholipase C (PLC) through G_q_ activation and internal Ca^2+^ release rather than the major PKC pathway [61]. In consistency, cAMP-independent activation of AQP2 involving intracellular Ca^2+^ release was shown in rat IMCD in a manner suggesting V2R action of phosphoinositide-specific PLC [52]. Also in favor of the PLC hypothesis, PKC has in several other studies been shown to enhance AQP2 endocytosis instead of apical trafficking. The underlying mechanism has been suggested to be mediated by actin cytoskeletal rearrangements and transient short-chain ubiquitination of AQP2 after either AVP withdrawal or activation of PKC independently of p(S256)-AQP2 abundance [62–65]. Thus, further investigations are needed to fully reveal the downstream mechanisms in AT1R-V2R crosstalk.

### Apical expressions of AQP2 and p-AQP2 in HF groups were increased

Additional immunohistochemistry revealed that all HF groups in our study developed marked apical labeling of AQP2 and p-AQP2 in IMCD compared to standard diet sham rats, despite decreased AT1R. Our study is consistent with previous results in mice lacking AT1_A_ receptors only in the collecting duct (CD-KO). When water deprived, these mice diminished AQP2 abundance but sustained almost complete apical targeting of IMCD AQP2 in response to AVP stimulation [49]. One possible explanation could be that ANG II-AT1R complexes situated in earlier nephron segments could modulate flow rate and tonicity of the tubular fluid entering the medullary collecting ducts, and thereby influence AQP2 trafficking. For example, hypotonicity favors internalization of p-AQP2 and AQP2 in cultured renal CD8cells, whereas hypertonicity enhances apical and basolateral AQP2 membrane accumulation in the IMCD [38,66–68]. Consistently, we have previously demonstrated that complete and global AT1R inhibition with candesartan reversed apical targeting of IMCD AQP2 and p-AQP2 in chronic HF rats [9]. Moreover, enhanced apical labeling of AQP2 and p-AQP2 in IMCD could also simply reflect increased AVP activity. Evidence of enhanced accumulation of cAMP in HF suggesting increased V2R activity has already been shown in Isolated IMCDs from HF rats and cardiomyopathic hamsters [46,69]. Recently, Brønd et al demonstrated that V2R-mediated cAMP accumulation was associated with elevated IM AQP2 protein expression and a fast recycling within 30 minutes of surface-associated V2R in HF rats, whereas the V2R in isolated IMCDs from control rats did not recycle to the receptor surface after AVP stimulation [46]. In another study by the same group using isolated medullary thick ascending limbs from HF rats, the enhanced AVP-V2R sensibility shown by cAMP accumulation was completely blocked by the AT1R blocker losartan [70]. Furthermore, administration of an angiotensin-converting enzyme inhibitor to HF rats normalized the increased AQP2 mRNA levels and restored GFR to sham levels [7,71]. These results are in line with our study, suggesting that ANG II is able to exert direct modulations on the V2R-mediated PKA pathway, resulting in enhanced apical targeting of total AQP2.

In the present study, HF rats spontaneously decreased receptor AT1R abundance at least in the IMCD, presumably as a result of high levels of circulating ANG II and enhanced AT1R activation. Even so, the rats preserved high levels of apical shuttling. Consistently, mice lacking the AT1R only in the collecting duct (CD-KO mice) showed substantial apical targeting when stimulated with DDAVP vs. controls. Interestingly, the CD-KO mice only reduced AT1_A_ mRNA levels by 43 ± 6.5% vs. controls in IMCD, indicating a residual amount of AT1R [8,9,49]. The observed differences in AQP2 targeting among the present and previous studies using AT1R blockage may be due to the quantity of still active AT1R in the IMCD in the CD-KO mice with a rather low threshold for potentiating AQP2 targeting [49]. Furthermore, the L-Sham+d rats displayed higher Gsα abundance than the L-HF+d rats, which could reflect differences in V2R activity. However, HF and L-HF rats also increased Gsα abundance vs. Sham and L-Sham (Fig. 5, *A* and *B*) similar to L-Sham+d (Fig. 5, *E*) but still failed to increase AQP2 levels. Based on these observations, we suggest that the effects of AT1R on total AQP2 levels in early-stage HF could be mediated by separate pathways independently of the V2R pathway, despite increased levels of V2R abundance. However, our study does not explain why the compensational effect on AQP2 is only effective when it comes to abundance and not shuttling in early-stage HF, which calls for further investigations.

### The (pro)-renin receptor was increased in L-HF

Apart from ANG I formation, (P)RR has been shown to induce intracellular signaling cascades involving the MAP kinases ERK1/2, which also have been suggested as a modulator of the V2R-cAMP dependent pathway in AQP2 regulation [72,73]. Shao and coworkers showed that long-term low sodium diet (13 days) increased plasma and intrarenal ANG II, renin and medullary renin mRNA, but failed to increase urinary excretion of angiotensinogen (AGT) and ANG II vs. standard diet rats [18]. Consistently, our study demonstrated that standard diet Sham rats and L-Sham rats had comparable levels of IM AT1R and (P)RR. In contrast, (P)RR was increased in L-HF but decreased in L-HF+d in the presence of lowered AT1R (Fig. 7, D). These data suggest that IM (P)RR is able to be regulated independently of AT1R but might be sensitive to circulating AVP levels, inner medullary osmolality, or both. Decreased AT1R and unaffected V2R levels despite of elevated (P)RR abundance in L-HF could also be part of an escape-mechanism which could be blunted in chronic HF under certain settings where the effect ANG II exceed the effect of AVP. Interestingly, AVP and oxytocin are shown to co-localize with (P)RR in the supraoptic and paraventricular nuclei in human and mouse hypothalamus [74,75]. Furthermore, a recent study using in situ hybridization for (P)RR mRNA and immunohistochemistry double-staining for the pituitary hormones showed that (P)RR mRNA was expressed in most of the GH cells and ACTH cells in the anterior lobe of the pituitary gland, strongly suggesting a central crosstalk between AVP release and (P)RR, but further investigation on this issue is demanded [76].

### AQP4 expression is increased in HF and L-HF rats but decreased in L-HF+d

The role IM AQP4 plays in the fine tuning of free water reabsorption is unclear. Whereas targeted disruption of AQP4 in mice results in a 75% reduction in the osmotic water permeability of the inner medullary collecting duct (IMCD), 48-h fluid restriction in rats did not affect IM AQP4 [12,77]. In contrary to former beliefs, AQP4 has been shown to be in part AVP-sensitive [78]. Van Hoek et. al. demonstrated that AQP4 subtype abundance could be increased by V2R in a PKA dependent manner [79]. Studies in brain and retina have indicated that AVP and RAS hormones could play an important role by increasing AQP4 abundance under certain pathophysiological conditions [78,80]. Consistently, AQP4 was increased in L-Sham+d, HF, L-HF, and L-Sham vs. Sham. These results indicate that ANG II also takes part in AQP4 regulation in the kidney. Water retention in combination with decreased GFR and severe hyponatremia in nephrotic syndrome and liver cirrhosis have been associated with downregulation of AQP4 [81–83]. L-HF+d rats, who exhibited the most severe hyponatremia and a major decrease in Ccr, correspond with these findings. Indeed, short-term liver cirrhosis studies have revealed that decreased AQP2 expression is possible in the presence of hyponatremia, hypoosmolality, and sustained elevated AVP [83–85]. Thus, in certain settings of extracellular fluid volume expansion, excessive water retention with hyponatremia can occur in the absence of increased AQP2 abundance. Terris et al. suggested that the underlying mechanism was different from that in AVP-escape, because AVP-escape only affects AQP2 abundance but not AQP3 and AQP4, and our present findings support this hypothesis [13,83]. However, we cannot fully explain the observed differences in AQP4 abundance from L-Sham+d and L-HF+d in this study. McCoy and coworkers demonstrated that AQP4 facilitated water permeability is regulated by protein kinase C (PKC) raising the question whether circulating ANG II through AT1R could be modulating AQP4 through AT1R-V2R crosstalk, despite of downregulated AT1R [9,55,86,87].

### AQP1 is downregulated in sodium restricted sham rats independently of DDAVP

The IMCD paracellular environment is highly hyperosmotic due to the reuptake and up-concentration of urea in this zone in conjunction with the thick ascending limbs, thereby enabling fine-tune urine concentration in the IMCD. This makes the inner medulla a focus for water retention states such as HF. Consistent with previous results in chronic-stage HF, AQP1, AQP3 and Na-K-ATPase were unaffected in HF vs. Sham [11]. Also, sodium restriction and DDAVP infusion did not affect AQP1 abundance among the HF group. In contrast, L-Sham and L-Sham+d downregulated IM AQP1 vs. Sham and L-HF rats. Decreased AQP1 in cortex and the inner stripe of outer medulla in sodium restricted rats co-treated with DDAVP and candesartan has previously been shown by our group [8]. It was suggested that AQP1 downregulation in these parts of the kidney could be due to either direct AT1R blockade or decreased GFR. In the present study, AT1R abundance was unaffected among sham groups (Fig. 6, *E*) and GFR (shown by Ccr) in L-Sham and Sham was unchanged. AQP1 alterations were confined to the sham groups only (Fig. 8, *A*, *B*, *D*, *E*). These observations make changes in GFR or AT1R as the prime effector less likely. Previous studies have demonstrated that AQP1 can be upregulated by elevated osmolality in kidney IM [88,89]. Urea, along with sodium chloride, constitutes a large portion of the medullary hyperosmolar driving force for water transport. U/P osmolality ratio increased in all animals receiving low sodium diet. Low sodium delivery to the loop of Henle and distal collecting ducts in combination with increased urea excretion could then lead to decreased medullary osmolality. These events could explain why AQP1 was decreased in L-Sham and L-Sham+d but they do not explain why AQP1 abundance in L-HF and L-HF+d was unaffected. The increased plasma urea in L-HF vs. L-Sham indicates that enhanced AVP-mediated urea reabsorption takes place in the terminal part of IMCD in HF. Hence, reduced IM osmolality as indicated by increased urea washout could be responsible for decreased AQP1 in L-Sham and L-Sham+d vs. Sham (Table 3) [43,90,91].

### Study limitations and clinical perspectives

The changes in protein abundance from the immunoblots in this study can seem relatively modest. However, the presented changes are within the range previously published using the same model [9,45,46]. In our experience, rats are resilient animals capable of surviving remarkably large infarcts with only modest changes in cardiac pumping ability and renal water and sodium retention, when compared to humans. Nevertheless, previous findings in HF rats have been confirmed in human studies [5,92,93]. We believe that our results are clinically important. They suggest that the kidney may possess intrinsic protecting factors against chronically elevated AVP during cardiac remodeling after MI, shown by partly blunted AQP2 recruitment. Blunted AQP2 recruitment has not previously been described in HF. Our results indicate that inhibition of AT1R synthesis is responsible. In the kidney, chronically RAS activation can lead to medullary necrosis, renal fibrosis, and chronic kidney failure [94]. Similar findings have been observed in the heart [95,96]. Further studies need to be performed to investigate whether targeted drug regimens can sustain or even enhance the beneficial compensation mechanism by the kidney seen in early-stage HF into later stages of HF. Finding the underlying reason why this protection seems to be stopping with finalization of cardiac remodeling needs to be further investigated and could have beneficial implication on the future clinical therapy post-MI. This could potentially lead to suppression of the vicious cycle of HF and/or decrease the levels of side effects in drug therapy such as drug induced hyponatremia.

## Summary and Conclusion

Early-stage HF rats developed hyponatremia, hypo-osmolality, and decreased Ccr, but exhibited comparable IM AQP2 and p-AQP2 abundance to sham groups, despite of increased V2R abundance and marked apical staining of AQP2 shown by immunocytochemistry. Decreased type-1_A_ angiotensin II receptor abundances in all HF groups likely play a role in the transduction of these effects. (P)RR in the HF groups altered independently of V2R and AT1R abundance. AQP4 was decreased in L-HF rats but increased in sodium restricted Sham when chronically infused with DDAVP. Sodium restriction elicited decreased AQP1 abundance in Sham rats, but not in HF rats. We suggest that during early-stage HF the kidney may possess intrinsic protecting features against chronically elevated AVP and sustained increased AQP2 abundance. In conclusion, this study supports the importance of V2R-AT1R crosstalk in the development of HF.
